# Identifying Cardiac Amyloid in Aortic Stenosis: ECV Quantification by CT in TAVR Patients

**DOI:** 10.1016/j.jcmg.2020.05.029

**Published:** 2020-10

**Authors:** Paul R. Scully, Kush P. Patel, Bunny Saberwal, Ernst Klotz, João B. Augusto, George D. Thornton, Rebecca K. Hughes, Charlotte Manisty, Guy Lloyd, James D. Newton, Nikant Sabharwal, Andrew Kelion, Simon Kennon, Muhiddin Ozkor, Michael Mullen, Neil Hartman, João L. Cavalcante, Leon J. Menezes, Philip N. Hawkins, Thomas A. Treibel, James C. Moon, Francesca Pugliese

**Affiliations:** a Barts Heart Centre, St. Bartholomew’s Hospital, London, United Kingdom; b Institute of Cardiovascular Sciences, University College London, London, United Kingdom; c Siemens Healthineers, Forchheim, Germany; d William Harvey Research Institute, Queen Mary University of London, London, United Kingdom; e John Radcliffe Hospital, Oxford University Hospitals, Oxford, United Kingdom; f Nuclear Medicine, Swansea Bay UHB, Port Talbot, United Kingdom; g Minneapolis Heart Institute, Minneapolis, Minnesota; h Institute of Nuclear Medicine, University College London, London, United Kingdom; i NIHR University College London Hospitals Biomedical Research Centre, London, United Kingdom; j National Amyloidosis Centre, University College London, London, United Kingdom; k NIHR Barts Biomedical Research Centre, London, United Kingdom

**Keywords:** aortic stenosis, cardiac amyloidosis, computed tomography, extracellular volume, AS, aortic stenosis, AS-amyloid, dual aortic stenosis and cardiac amyloid pathology, ATTR-CA, transthyretin-related cardiac amyloidosis, AUC, area under the curve, CT, computed tomography, CTCA, computed tomography coronary angiogram, DPD, ^99m^Tc-3,3-diphosphono-1,2-propanodicarboxylic acid, ECG, electrocardiogram, ECV, extracellular volume, ECV_CT_, extracellular volume quantification by computed tomography imaging, hs-TnT, high-sensitivity troponin T, IVSd, interventricular septal diameter, MCF, myocardial contraction fraction, PWd, posterior wall diameter, RBBB, right bundle branch block, SPECT, single-photon emission computed tomography, TAVR, transcatheter aortic valve replacement

## Abstract

**Objectives:**

The purpose of this study was to validate computed tomography measured ECV (ECV_CT_) as part of routine evaluation for the detection of cardiac amyloid in patients with aortic stenosis (AS)-amyloid.

**Background:**

AS-amyloid affects 1 in 7 elderly patients referred for transcatheter aortic valve replacement (TAVR). Bone scintigraphy with exclusion of a plasma cell dyscrasia can diagnose transthyretin-related cardiac amyloid noninvasively, for which novel treatments are emerging. Amyloid interstitial expansion increases the myocardial extracellular volume (ECV).

**Methods:**

Patients with severe AS underwent bone scintigraphy (Perugini grade 0, negative; Perugini grades 1 to 3, increasingly positive) and routine TAVR evaluation CT imaging with ECV_CT_ using 3- and 5-min post-contrast acquisitions. Twenty non-AS control patients also had ECV_CT_ performed using the 5-min post-contrast acquisition.

**Results:**

A total of 109 patients (43% male; mean age 86 ± 5 years) with severe AS and 20 control subjects were recruited. Sixteen (15%) had AS-amyloid on bone scintigraphy (grade 1, n = 5; grade 2, n = 11). ECV_CT_ was 32 ± 3%, 34 ± 4%, and 43 ± 6% in Perugini grades 0, 1, and 2, respectively (p < 0.001 for trend) with control subjects lower than lone AS (28 ± 2%; p < 0.001). ECV_CT_ accuracy for AS-amyloid detection versus lone AS was 0.87 (0.95 for ^99m^Tc-3,3-diphosphono-1,2-propanodicarboxylic acid Perugini grade 2 only), outperforming conventional electrocardiogram and echocardiography parameters. One composite parameter, the voltage/mass ratio, had utility (similar AUC of 0.87 for any cardiac amyloid detection), although in one-third of patients, this could not be calculated due to bundle branch block or ventricular paced rhythm.

**Conclusions:**

ECV_CT_ during routine CT TAVR evaluation can reliably detect AS-amyloid, and the measured ECV_CT_ tracks the degree of infiltration. Another measure of interstitial expansion, the voltage/mass ratio, also performed well.

Aortic stenosis (AS) is the most common valve disease in the developed world (1). Its prevalence increases with age, with 2.8% to 4.8% of patients ≥75 years of age having at least moderate AS (2,3). Once symptomatic with severe AS, outcomes are poor without intervention (4), which can be either surgical or transcatheter aortic valve replacement (TAVR). TAVR numbers are increasing fast worldwide, in response to both an aging population and technological developments (5,6).

Another disease of aging is wild-type transthyretin-related cardiac amyloidosis (ATTR-CA); deposits are present within the myocardium at autopsy in up to 25% of patients ≥85 years of age (7). Recent work has shown a remarkably high prevalence (14% to 16%) of ATTR-CA in the elderly AS population being considered for TAVR (AS-amyloid) (8,9). We do not yet fully understand the significance of this dual pathology, either for valve intervention or the role for specific amyloid therapies such as tafamidis (10), patisiran (11), and inotersen (12), but detection is likely to be important. Conventional first-line investigations for ATTR-CA, such as echocardiography, blood biomarkers, or electrocardiogram (ECG), are confounded by the dual pathology. ATTR-CA can now be diagnosed noninvasively by using bone scintigraphy, such as ^99m^Tc-3,3-diphosphono-1,2-propanodicarboxylic acid (DPD), ^99m^Tc-pyrophosphate, and ^99m^Tc-hydroxymethylene diphosphonate, coupled with a negative search for a plasma cell dyscrasia (13). Although availability and awareness are increasing, it requires an extra test in elderly, often frail, patients.

As part of routine TAVR evaluation, patients typically undergo contrast computed tomography (CT) imaging to assess annulus dimensions, coronary artery height (and patency, where possible), and vascular access. Contrast CT imaging can also be used to measure the myocardial extracellular volume (ECV) in a manner similar to cardiovascular magnetic resonance (CMR) (14,15). The ECV increases moderately with diffuse fibrosis but massively with amyloidosis (16). Our group has previously validated ECV quantification by CT imaging (ECV_CT_) against CMR and histology (endomyocardial biopsy) in severe AS (17,18) and against CMR in cardiac amyloid (18). Unlike recommended CMR acquisition, the ECV_CT_ acquisition for cardiac amyloid can be performed earlier at 5 min rather than 10 min post-contrast (18).

In the current study, we hypothesized that ECV_CT_ as part of routine TAVR evaluation CT imaging would be able to detect AS-amyloid. To improve workflow, we also sought to optimize the scanning protocol in terms of dose and timing (shortened scan delay).

## Methods

This work represents a prespecified analysis of a subset of patients of the ATTRact-AS study (Role of Occult Cardiac Amyloid in the Elderly With Aortic Stenosis; NCT03029026). Relevant local ethics approvals were obtained. Patients ≥75 years of age with severe AS referred for TAVR at Barts Heart Centre (London, United Kingdom) and undergoing CT imaging as part of their clinical evaluation were included in this substudy. The only exclusion criterion was being unable to provide informed consent.

Patients underwent routine clinical TAVR evaluation, including baseline ECG, echocardiography, and CT imaging. The additional research procedures were DPD scintigraphy (before TAVR), the additional CT acquisitions for ECV_CT_, and, if not already performed, contemporaneous blood tests for hematocrit, high-sensitivity troponin T (hs-TnT), and N-terminal pro–B-type natriuretic peptide. Twenty control patients also underwent ECV_CT_. These subjects were recruited for a separate study evaluating ECV_CT_ in patients with suspected coronary artery disease, and all had contemporary CMR showing normal biventricular size and function with no late gadolinium enhancement. These control patients were included to provide an estimate of “normal” ECV_CT_ and were not used in the screening calculations.

### Electrocardiogram

As we have described previously (19), Sokolow-Lyon criteria were calculated as the sum of the amplitude of the S-wave in lead V_1_ and the R-wave in lead V_5_ or V_6_ (whichever was greater) (20). The voltage/mass ratio was defined as the Sokolow-Lyon total divided by the indexed left ventricular (LV) mass on echocardiography. Patients with bundle branch block or a ventricular paced rhythm were excluded from this analysis (21). Low limb lead voltages were defined as all limb leads with an amplitude ≤0.5 mV.

### Echocardiography

AS severity (aortic valve peak velocity, mean gradient, and valve area), biventricular systolic and left ventricular diastolic function were assessed using transthoracic echocardiography (22, 23, 24, 25, 26). As we have described previously (19), LV ejection fraction was calculated using Simpson’s biplane if possible (otherwise visually) and the indexed stroke volume was calculated using the LV outflow tract velocity time integral and diameter, which was then indexed to body surface area. Relative wall thickness was defined as: (2 × posterior wall diameter)/(LV internal diameter at end-diastole) (25). LV mass was calculated by using the formula from Devereux et al. (26):

$$\text{LV mass}=0.8\times 1.04\times {\left[\left(\text{IVSd }+\text{ LVIDd }+\text{ PWd}\right)\right]}^{3}-{\text{LVIDd}}^{3}]+0.6$$

where *IVSd* is the interventricular septal diameter, *LVIDd* is the LV internal dimension at end-diastole, and *PWd* is the posterior wall diameter. Longitudinal strain analysis was performed off-line by an accredited echocardiographer using 2-D Cardiac Performance Analysis software (TomTec Imaging Systems GmbH, Unterschleissheim, Germany).

In view of the fact AS and amyloid may have myocardial impairment better captured by myocardial contraction fraction (MCF = stroke volume/myocardial volume) (27), we calculated this with LV end-diastolic volume as 4.5 × LVIDd^2^; LV end-systolic volume as 3.72 × LVIDs^2^; stroke volume as LV end-diastolic volume − LV end-systolic volume; LV mass as 1.04 × [(IVSd + LVIDd + PWd)^3^ − LVIDd^3^]; and the myocardial volume as the LV mass/mean density of myocardium (1.04 g/ml).

### DPD Scintigraphy

All DPD scans were performed by using either a hybrid single-photon emission CT (SPECT)/CT gamma camera (Philips BrightView, Blue Bell, Pennsylvania) or a SPECT gamma camera (Symbia, Siemens Healthineers USA, Malvern, Pennsylvania) following the injection of 700 MBq DPD. The imaging protocol consisted of an early and late (5 min and 3 h, respectively) planar whole-body image, with a SPECT/CT scan or SPECT scan only of the chest at 3 h. DPD scans were reported by 2 experienced clinicians using the Perugini grading system (28), with grade 0 being negative and grades 1 to 3 increasingly positive. DPD scan findings were independently reviewed by the National Amyloidosis Centre (London, United Kingdom). All patients with a positive DPD scan were discussed with the managing clinicians and, where appropriate, referred to the National Amyloidosis Centre for further review.

### CT Scans

**Figure 1:**
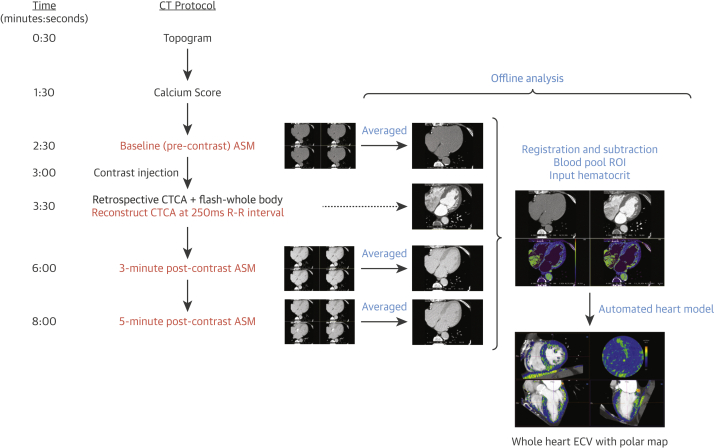
ECV_CT_ Protocol and Offline Analysis Integrated Into TAVR Planning Cardiac CT Text in **red** represents additional image acquisition/reconstruction in scanning protocol for the extracellular volume quantification by computed tomography (ECV_CT_). Text in **blue** represents steps in off-line analysis. ASM = axial shuttle mode; CT = computed tomography; CTCA = computed tomography coronary angiography; ECV = extracellular volume; ROI = region of interest.

All CT scans were performed on a Somatom FORCE scanner (Siemens Healthineers, Erlangen, Germany). The TAVR evaluation CT protocol at Barts Heart Centre involves a topogram, calcium score, timing bolus, gated CT coronary angiogram (CTCA) acquired retrospectively, and a FLASH whole-body scan (lung apices down to the lesser trochanters). The total volume of Omnipaque 300 (iohexol) contrast (GE Healthcare, Chicago, Illinois) was fixed at 90 ml (including the 10 ml timing bolus) for the clinical scan, with no additional contrast used for research purposes. The additional acquisitions for research were a baseline “axial shuttle mode” pre-contrast after the calcium score and further pseudo-equilibrium axial shuttle mode datasets, both triggered 250 ms after the R-wave, at 3 and 5 min post-contrast (following the FLASH whole-body scan). All axial shuttle mode datasets (4 repetitions every other heartbeat, single breath hold) were acquired at a fixed tube voltage of 80 kV and tube current-time product of 370 mA. Image reconstruction was performed by using the same field of view in all 3 datasets. An additional dataset was reconstructed from the retrospectively acquired CTCA at 250 ms of the R-R interval, with a field of view matching that of the axial shuttle mode datasets (Figure 1) to be used as a landmark for ECV_CT_ measurement and overlay.

### ECV Analysis

**Figure 2:**
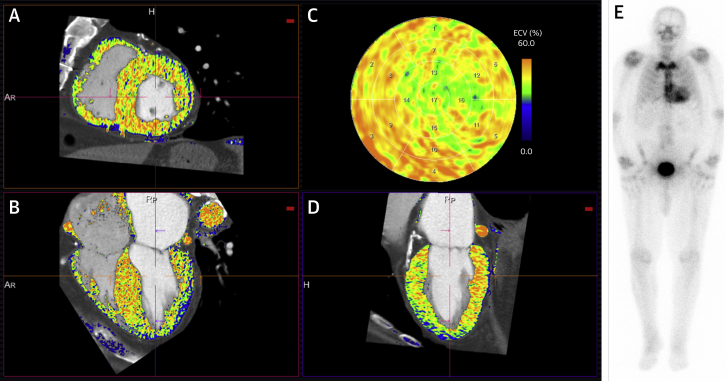
Automated **ECV_CT_** Heart Model Output With Corresponding 3-h Planar DPD Image ECV_CT_ map superimposed on the CTCA images **(A to D)** and corresponding 3-h planar DPD image **(E)**. The endocardial and epicardial contours can be edited in the short-axis **(A)**, 4-chamber **(B)**, and 2-chamber **(D)** views to produce an ECV_CT_ American Heart Association 17-segment polar map **(C)**. This is a patient with aortic stenosis (AS) amyloid (Perugini grade 2 ^99m^Tc-3,3-diphosphono-1,2-propanodicarboxylic acid [DPD] scintigraphy) with total myocardial ECV_CT_ is globally elevated at 47%. Abbreviations as in Figure 1.

We have briefly described this technique previously (29). Nonrigid registration software (Hepacare, Siemens Healthineers) allowed averaging and aligning of the axial shuttle mode datasets to improve image quality and reduce noise. The averaged baseline image was then subtracted from the averaged 3- and 5-min post-contrast images (providing a partition coefficient) and then registered with the CTCA image. A region of interest was placed in the LV blood pool on the CTCA image and the hematocrit (usually taken on the same day) inputted, generating a myocardial ECV_CT_ map via the formula: ECV_CT_ = (1 − hematocrit) × (ΔHU_myo_ /ΔHU_blood_), where ΔHU is the change in Hounsfield unit attenuation pre-contrast and post-contrast (i.e., HU_post-contrast_−HU_pre-contrast_) (18,30,31). This information was loaded into prototype software (Cardiac Function, Siemens Healthineers), which allowed the ECV_CT_ map to be superimposed on the CTCA image, the myocardial contours to be edited, and the results to be displayed as a 17-segment polar map (Figures 1 and 2). When calculating total ECV_CT_, focally elevated ECV_CT_ (e.g., likely myocardial infarction) were not excluded, but American Heart Association segments with significant beam-hardening artifacts from adjacent pacing wires (n = 4) were excluded. LV mass was calculated using the standard automated software on clinical syngo.via (Siemens Healthineers) workstations.

### Statistical analysis

Statistical analysis was performed by using IBM SPSS Statistics version 25 (IBM SPSS Statistics, IBM Corporation, Armonk, New York) software. Where appropriate, results are described as mean ± SD or median (interquartile range). Kruskal-Wallis analysis of variance was used when comparing >2 groups as the omnibus test, with the Dunn-Bonferroni test for pairwise comparison. Bland-Altman analysis was performed to compare 3- and 5-min post-contrast time points, as well as the impact of dose reduction. Receiver-operating characteristic curve analysis was used to assess diagnostic performance. Student’s *t*-test or the Mann-Whitney *U* test was used to compare continuous variables and either chi-squared or Fisher exact testing for categorical data was used as appropriate. Univariate and multivariate analyses were performed by using binary logistic regression, with the presence of AS-amyloid as the dependent variable. Variables for the multivariate analysis were selected based on statistical significance on univariate analysis and clinical relevance, while avoiding multicollinearity (e.g., only 1 parameter reflecting LV mass was included). Variance inflation factors for each independent variable used in the multivariate analysis were calculated as one divided by the tolerance (defined as 1 − R^2^ of the regression model for the studied variable). The voltage/mass ratio was not included in the multivariate analysis to avoid excluding nearly one-third of patients (32 in total) with bundle branch block or ventricular paced rhythm. The DeLong test was used to compare areas under the curves (AUCs). A 2-sided p value <0.05 was considered statistically significant.

## Results

A total of 109 patients (43% male; mean age 86 ± 5 years) with severe AS were included in this substudy of ATTRact-AS. Overall, LV ejection fraction was 54 ± 10%, peak aortic valve velocity was 4.1 ± 0.6 m/s, the mean pressure gradient was 41 ± 14 mm Hg, and the aortic valve area was 0.71 ± 0.23 cm^2^. Patient characteristics (demographics, comorbidities, ECG, echocardiography, CT scan, and blood test results) are described in Table 1. As might be expected, hypertension, hypercholesterolemia, diabetes mellitus, and atrial fibrillation were common in this group of patients. Venous hematocrit was 0.38 ± 0.04, which was usually taken on the same day as the CT scan (median 0 days; interquartile range 0 to 22 days). Twenty control subjects were also recruited separately to provide an idea of “normal” ECV_CT_ (65% male; mean age 60 ± 11 years).

**Table 1:** Basic Demographic Characteristics and Clinical, Echocardiography, and Computed Tomography Parameters for Patients With Lone AS and AS-Amyloid

	Overall (N = 109)	Lone AS (n = 93)	AS-Amyloid (n = 16)	p Value
Demographic characteristics				
Male	47 (43)	38 (41)	9 (56)	0.25
Age (yrs)	86 ± 5	85 ± 5	88 ± 5	0.08
Clinical parameters				
Hypertension	86 (79)	73 (78)	13 (81)	1.00
Hypercholesterolemia	44 (40)	37 (40)	7 (44)	0.77
Diabetes mellitus	25 (23)	24 (26)	1 (6)	0.11
Atrial fibrillation	49 (45)	41 (44)	8 (50)	0.66
Permanent pacemaker	14 (13)	12 (13)	2 (13)	1.00
ECG parameters				
Heart rate (beats/min)	73 ± 15	73 ± 16	70 ± 14	0.46
Low-voltage limb leads	1 (1)	1 (1)	0 (0)	1.00
S-L criteria (mV)	2.5 ± 1.0	2.6 ± 1.0	1.8 ± 0.5	**0.048**
First-degree HB∗	21 (19)	20 (22)	1 (7)	0.30
QRS duration (ms)	106 ± 25	103 ± 26	120 ± 20	**0.01**
LBBB∗	10 (10)	8 (9)	2 (13)	1.00
RBBB∗	12 (12)	6 (7)	6 (38)	**0.002**
Echocardiogram parameters				
Left ventricle				
LVEF (%)	54 ± 11	54 ± 10	58 ± 10	0.18
Indexed SV (ml/m^2^)	38 ± 11	38 ± 12	35 ± 9	0.29
IVSd (cm)	1.3 ± 0.2	1.3 ± 0.2	1.4 ± 0.3	**0.002**
PWd (cm)	1.1 ± 0.3	1.1 ± 0.2	1.3 ± 0.3	**<0.001**
Relative wall thickness (cm)	0.50 ± 0.15	0.48 ± 0.13	0.61 ± 0.20	**0.002**
Indexed LV mass (g/m^2^)	116 ± 37	113 ± 37	137 ± 31	**0.01**
MCF (%)	23.7 ± 8.4	24.5 ± 8.4	19.4 ± 7.2	**0.02**
Mitral annulus S′ (m/s)	0.06 ± 0.01	0.06 ± 0.01	0.05 ± 0.01	0.08
Global LV LS (%)	–15 ± 6	–15 ± 7	–16 ± 6	0.62
Diastolic function				
E/A ratio	0.8 (0.7–1.3)	0.8 (0.7–1.1)	1.4 (0.9–2.3)	0.07
Lateral E/E′	17 ± 10	17 ± 8	21 ± 15	0.28
MV deceleration time (ms)	235 ± 90	234 ± 92	238 ± 80	0.87
LA diameter (cm)	4.1 ± 0.7	4.0 ± 0.7	4.4 ± 0.6	0.08
RV function				
TAPSE (cm)	1.91 ± 0.46	1.92 ± 0.48	1.89 ± 0.36	0.82
AV				
Peak velocity (m/s)	4.10 ± 0.63	4.12 ± 0.63	4.02 ± 0.62	0.55
Mean gradient (mm Hg)	69 ± 21	42 ± 14	38 ± 12	0.36
AVA (cm^2^)	0.71 ± 0.23	0.71 ± 0.23	0.72 ± 0.21	0.92
CT parameters				
AV calcium score (HU)	2,115 (1,497–3,184)	2,107 (1,491–3,109)	2,170 (1,665–3,602)	0.60
Indexed LV mass (g/m^2^)	74 ± 19	72 ± 17	91 ± 24	0.01
Composite parameters				
V/M ratio (mV/g/m^2^)	0.025 ± 0.01	0.026 ± 0.011	0.013 ± 0.004	**<0.001**
Blood results				
Hematocrit	0.38 ± 0.04	0.38 ± 0.04	0.38 ± 0.05	0.92
Creatinine (mmol/l)	108 ± 38	106 ± 37	120 ± 38	0.16
eGFR (ml/min/1.73 m^2^)	53 ± 16	54 ± 17	47 ± 12	0.12
hs-TnT (ng/l)	34 (15–38)	20 (14–34)	43 (28–75)	**0.001**
NT-proBNP (ng/l)	1,517 (671–3,703)	1,361 (593–2,816)	3,668 (1,259–5,165)	**0.03**

Values are n (%), mean ± SD, or median (interquartile range).

AV = aortic valve; AVA = aortic valve area; HB = heart block; E/A = early to atrial wave ratio; eGFR = estimated glomerular filtration rate; hs-TnT = high-sensitivity troponin T; HU = Hounsfield units; IVSd = interventricular septum diameter; LA = left atrial; LBBB = left bundle branch block; LS = longitudinal strain; LV = left ventricular; LVEF = left ventricular ejection fraction; MCF = myocardial contraction fraction; MV = mitral valve; NT-proBNP = N-terminal pro–B-type natriuretic peptide; PWd = posterior wall diameter; RBBB = right bundle branch block; S-L = Sokolow-Lyon criteria; SV = stroke volume; TAPSE = tricuspid annular plane systolic excursion; V/M = voltage mass ratio.

∗ Missing electrocardiogram (ECG) data in 4 lone aortic stenosis (AS) patients and 1 AS-amyloid patient; percentages and statistics quoted reflect this.

### Detection of AS-amyloid

In this substudy, 16 patients (15%) had AS-amyloid diagnosed according to bone scintigraphy (grade 1, n = 5; grade 2, n = 11); their average age was 88 ± 5 years, and 56% were male. A plasma cell dyscrasia was detected in 6 patients (38%), who were either referred to the National Amyloidosis Centre or reviewed with the clinical team, and light-chain (AL) amyloid was believed unlikely in all cases. All patients genotyped so far (n = 9 [56%]) were wild type.

There was no difference in the age (88 ± 5 years vs. 85 ± 5 years; p = 0.08) or proportion of male patients (56% vs 41%; p = 0.25) when comparing patients with AS-amyloid versus those with lone AS. The cardiovascular risk profile (hypertension, hypercholesterolemia, and diabetes mellitus), presence of AF, or permanent pacemaker pre-procedure were similar. Patients with AS-amyloid had a longer QRS duration and higher prevalence of right bundle branch block (RBBB), as well as lower ECG voltage according to Sokolow-Lyon criteria and lower voltage/mass ratio. In AS-amyloid, parameters reflecting LV thickness and mass were higher, whereas the MCF was lower. Global longitudinal strain was impaired in both AS-amyloid and lone AS but did not differ. Both hs-TnT and N-terminal pro–B-type natriuretic peptide levels were higher in AS-amyloid (Table 1).

#### ECV_CT_ findings

**Figure 3:**
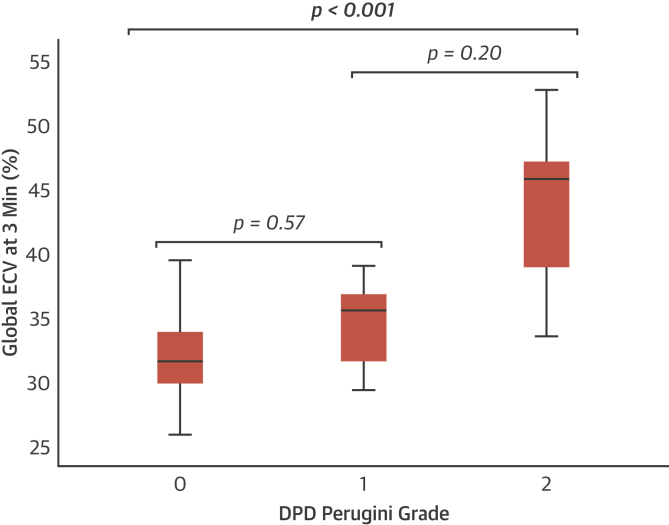
Box and Whisker Plot Showing the Variation in ECV_CT_ Between DPD Perugini Grades p < 0.001 for trend and for the pairwise comparison of grade 0 versus grade 2. Abbreviations as in Figures 1 and 2.

**Figure 4:**
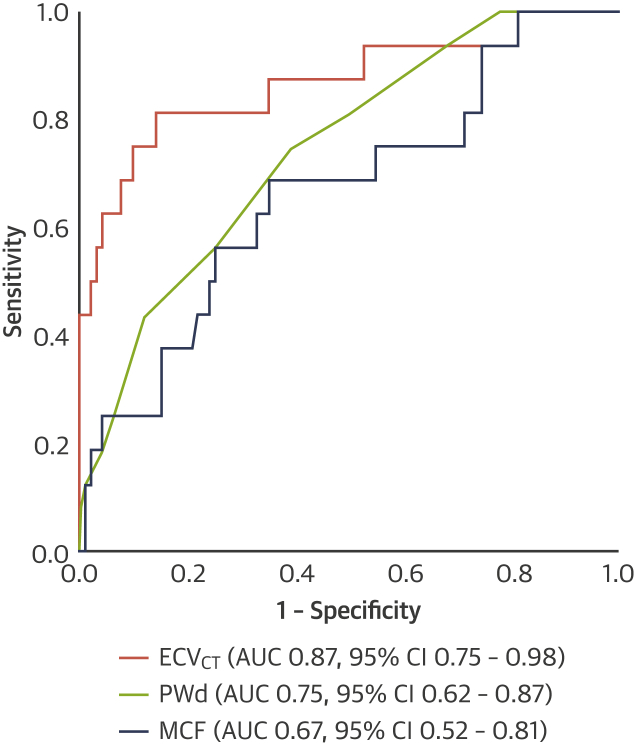
Receiver-Operating Characteristic Curve for the Detection of Any Cardiac Amyloid (DPD Perugini Grade 1 or 2) Using ECV_CT_ With a 3-Min Post-Contrast Acquisition, PWd, and MCF The voltage/mass ratio was not included because this approach would have excluded nearly one-third of patients (32 in total) due to bundle branch block or ventricular paced rhythm. AUC = area under the curve; CI = confidence interval; MCF = myocardial contraction fraction; PWd = posterior wall diameter; other abbreviations as in Figure 1.

ECV_CT_ was feasible for measurement in all patients for whom data were obtained. ECV_CT_ was 32 ± 3%, 34 ± 4%, and 43 ± 6% in those patients with Perugini grades 0, 1, and 2, respectively, using a 3-min post-contrast acquisition (p < 0.001 for trend) (Figure 3, Central Illustration). By comparison, ECV_CT_ in control subjects was 28 ± 2% using a 5-min post-contrast protocol, lower than in those patients with lone AS at similar post-contrast timing (33 ± 4%; p < 0.001). For the detection of any cardiac amyloid in patients with AS (DPD Perugini grade 1 or 2), the AUC was 0.87 (95% confidence interval: 0.75 to 0.98) using a 3-min post-contrast acquisition (Figure 4). Different ECV_CT_ thresholds could be set: 29.2% (sensitivity 100%, specificity 19%, negative predictive value 100%); 31.4% (sensitivity 94%, specificity 48%, negative predictive value 98%); or 33.4% (sensitivity 88%, specificity of 66%, negative predictive value 97%). If repeated for the detection of only grade 2 AS-amyloid (because there is more uncertainty about the clinical significance of a Perugini grade 1 DPD), the AUC improved to 0.95 (95% confidence interval: 0.89 to 1.00), and an ECV_CT_ of 33.4% offered 100% sensitivity and 64% specificity, with a negative predictive value of 100%.

**Central Illustration:**
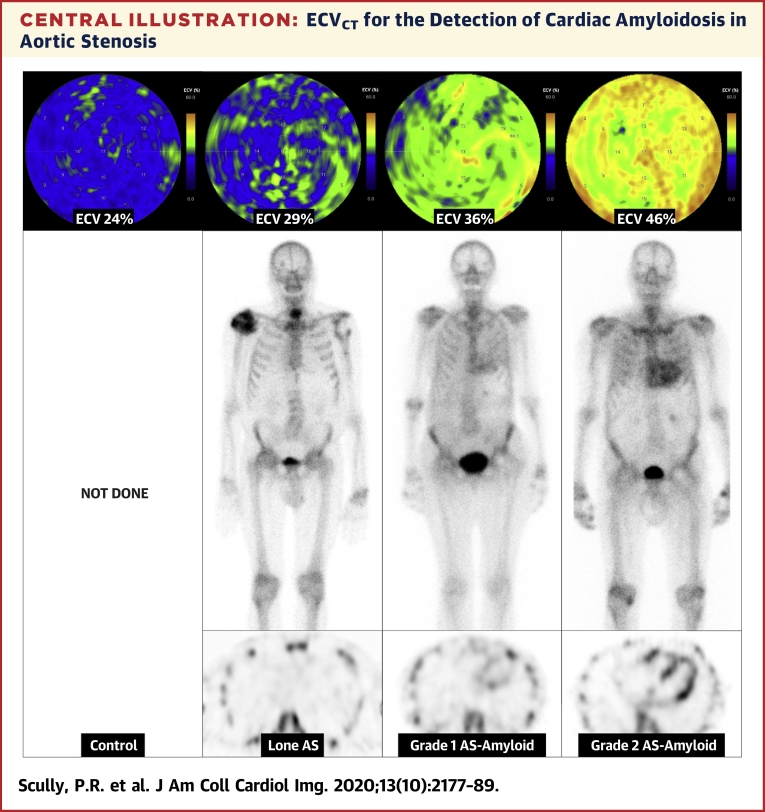
ECV_CT_ for the Detection of Cardiac Amyloidosis in Aortic Stenosis Extracellular volume (ECV) quantification by computed tomography (ECV_CT_) polar maps **(top)**, ^99m^Tc-3,3-diphosphono-1,2-propanodicarboxylic acid (DPD) planar **(middle)**, and axial single-photon emission computed tomography images **(bottom)** from control **(far left)** through lone aortic stenosis (AS), DPD Perugini grade 1, and DPD Perugini grade 2 **(far right)**.

#### Combined parameters

The voltage/mass ratio was lower in AS-amyloid and performed similar to ECV_CT_ for the detection of any cardiac amyloid (AUC: 0.87) but not as well for the detection of DPD grade 2 cardiac amyloidosis (AUC: 0.85). However, nearly one-third of patients (32 in total) had to be excluded from this analysis due to the presence of bundle branch block or a ventricular paced rhythm. MCF also performed reasonably well as a screening tool for any cardiac amyloid (AUC: 0.67), similar to PWd (AUC: 0.75; p = 0.12) but not as well as ECV_CT_ (AUC: 0.87; p = 0.003) (Figure 4).

#### Predictors of amyloid presence

Univariate analysis identified ECV_CT_, the presence of RBBB, and parameters associated with LV wall thickness or mass (IVSd, PWd, indexed LV mass, MCF, and voltage/mass ratio) as predictors of AS-amyloid (Table 2). Multivariate analysis of age, ECV_CT_, male sex, PWd, and RBBB showed that only ECV_CT_ and the presence of RBBB was associated with AS-amyloid (p = 0.001 and p = 0.01, respectively). For every 1% increase in ECV_CT_, there was a 1.6-fold increase in the likelihood of AS-amyloid (95% confidence interval: 1.21 to 2.10). Variance inflation factors for each multivariable were all close to 1, suggesting little multicollinearity (Supplemental Table 1).

**Table 2:** Univariate and Multivariate Binary Logistic Regression Analysis

	Univariate Analysis		Multivariate Analysis		
	p Value	Exp (B)	p Value	Exp (B)	95% CI for Exp (B)
Age (per yr increase)	0.08	1.10	0.38	1.09	0.90–1.30
ECV_CT_ (per % increase)	**<0.001**	1.49	**0.001**	1.60	1.21–2.10
AVA (per cm^2^ increase)	0.92	1.12	–		
AV mean gradient (per mm Hg decrease)	0.36	0.98	–		
AV V_max_ (per m/s decrease)	0.55	0.77	–		
AV calcium score (per HU increase)	0.56	1.00	–		
E/A ratio (per U increase)	0.04	1.74	–		
Male	0.26	1.86	0.81	0.81	0.14–4.60
GLS (per % decrease)	0.61	0.98	–		
hs-TnT (per ng/l increase)	0.06	1.01	–		
Indexed LV mass on echo (per g/m^2^ increase)	**0.02**	1.02	–		
Indexed SV (per ml/m^2^ decrease)	0.28	0.97	–		
IVSd (per cm increase)	**0.005**	44.66	–		
LA diameter (per cm increase)	0.08	2.04	–		
Lateral E/E′ (per U increase)	0.11	1.04	–		
LBBB	0.60	1.56	–		
LVEF (per % increase)	0.18	1.04	–		
MCF (per % decrease)	**0.02**	0.91	–		
Mitral annulus S′ (per m/s decrease)	0.08	0.00	–		
MV Dec time (per ms increase)	0.87	1.00	–		
NT-proBNP (per ng/l increase)	0.41	1.00	–		
PWd (per cm increase)	**0.003**	53.83	0.46	4.04	0.10–162.36
RBBB	**0.001**	9.22	**0.01**	16.84	1.87–148.54
RWT (per cm increase)	**0.006**	178.47	–		
S-L criteria (per mV decrease)	0.06	0.26	–		
TAPSE (per cm decrease)	0.81	0.87	–		
V/M ratio (per mV/g/m^2^ decrease)	**0.02**	0.00	–		

ECV_CT_ and the presence of RBBB were associated with AS-Amyloid on univariate and multivariate analysis. For every 1% increase in extracellular volume quantification by computed tomography imaging (ECV_CT_), there was a 1.6-fold increased likelihood of AS-amyloid. The V/M ratio was not included in the multivariate analysis because this would have excluded nearly one-third of patients (32 in total) due to bundle branch block or ventricular paced rhythm. Only 1 parameter representing LV wall thickness or mass was included in the multivariate analysis to avoid multicollinearity (in this case, PWd, as it had the strongest association on univariate analysis).

Exp (B) = exponentiation of the B coefficient; GLS = global longitudinal strain; MV = mitral valve; RWT = relative wall thickness; other abbreviations as in Table 1.

### Protocol optimization

A total of 104 patients completed both 3- and 5-min post-contrast acquisitions. The 3-min acquisition resulted in an acceptable ECV_CT_ result with very little bias; that is, 0.68 ± 1.2% lower than the 5-min acquisition (Supplemental Figure 1A). This bias appeared to increase above an ECV_CT_ of 40%, where such increases would not alter diagnostic accuracy.

### Dose reduction strategy

The dose length product for the full baseline and 3- and 5-min axial shuttle mode datasets was 182 ± 26 mGy·cm, 183 ± 24 mGy·cm, and 180 ± 24 mGy·cm, respectively. To investigate dose reduction strategies, we reanalyzed ECV_CT_ derived by using fewer shuttles (1 or 2 vs. 4) for the baseline and 3-min post-contrast acquisitions to assess any possible impact on diagnostic accuracy. Including 13 patients with lone AS and 14 patients with cardiac amyloid (grade 2, n = 9), there was minimal bias for 1 versus 4 shuttles (0.85 ± 2.1%) or 1 versus 2 shuttles (0.58 ± 1.47%) (Supplemental Figure 1B). Two outliers with differences beyond the 95% limits of agreement were patients both weighing >90 kg, for whom dose modulation would likely be used clinically. Reducing the protocol to a single shuttle pre-contrast and 3-min post-contrast reduces the dose by a factor of 4 (total dose length product of ∼90 mGy·cm, effective dose 2.3 mSv, using the higher cardiac k-factor of 0.026) (32).

## Discussion

ECV_CT_ can reliably detect dual AS-amyloid pathology in potential TAVR patients, with only an additional 3 min on top of the standard CT imaging evaluation and a small radiation burden (∼2.3 mSv), with measured ECV_CT_ not just detecting but tracking the degree of infiltration.

The ability to detect ATTR-CA noninvasively using bone scintigraphy has led to the increased realization that particularly wild-type ATTR-CA is not rare in the elderly. Recent research has shown just how common it is in elderly subjects with AS (8,9,33,34), but it is not limited to this population; indeed, 13% of patients with heart failure with preserved ejection fraction may have underlying cardiac amyloid (35), and 5% of those with LV hypertrophy may have variant ATTR-CA (this study used genotyping to screen LV hypertrophy patients and thus will have missed those with wild-type ATTR-CA) (36). The clinical impact of myocardial amyloid deposition in these patients with AS, however, remains unclear. We know that there may be a long preclinical phase and that prevalence increases with age, becoming the primary cause of death in supercentenarians (37). The spectrum therefore potentially extends from “bystander” to the primary cause of symptoms and adverse outcome, depending on the time of diagnosis and the myocardial tolerance. In turn, these are likely to be affected by amyloid burden, rate of amyloid deposition, the ability of the myocardium to adapt, and other myocardial “hits” such as, in this case, the increased afterload from AS. These may not be independent (the prevalence of AS-amyloid seems to be higher than what would be expected from age alone, suggesting that there may be an interaction), with an increased likelihood of amyloid in the interstitium of myocardium with afterload. This uncertainty of significance cascades into our terminology, which is not fixed. Should this be AS-amyloid or amyloid-AS? Similarly, is it cardiac amyloidosis (implies pathological) or cardiac amyloid (might be bystander deposition)? Here we have chosen AS-amyloid. These questions are about to become nonacademic and pressingly so, with the availability of 3 novel, potential, but costly medical therapies for cardiac amyloidosis (10, 11, 12) that have yet to be validated in patients with AS-amyloid. Clearly, an individualized treatment strategy is going to be needed, and answers will hopefully prove more forthcoming with the increasing availability of bone scintigraphy that will enable increased diagnostic rates and research activity.

The fact that pre-existing RBBB is associated with cardiac amyloidosis is intriguing and may prove relevant in the TAVR cohort given that we know RBBB is associated with a higher likelihood of post-TAVR pacemaker implantation (38) and worse outcomes (38,39). Although the authors did not investigate for the presence of concomitant cardiac amyloidosis, it is possible that the presence of RBBB at baseline might be an ominous sign that deserves further investigation.

**Figure 5:**
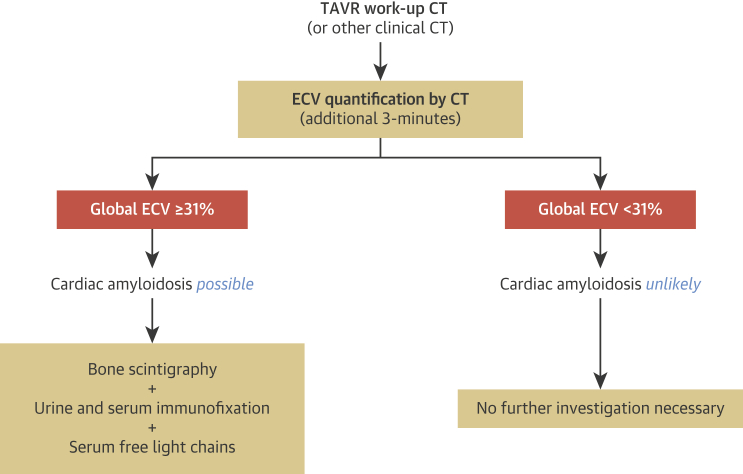
Proposed ECV_CT_ Screening Algorithm for Incorporation Into Routine Clinical Workflow The algorithm can be adjusted to an ECV_CT_ threshold of ≥29% for the detection of all grade 1 DPD patients. TAVR = transcatheter aortic valve replacement; other abbreviations as in Figures 1 and 2.

We propose CT imaging as a technique to increase AS-amyloid detection and present a diagnostic algorithm (Figure 5). Because ECV_CT_ is easy to implement, and the patient is already in the CT scanner, we think adoption of this technique could be high. This algorithm still uses bone scintigraphy (and exclusion of light-chain [AL] amyloid by serum free light chains, and serum and urine immunofixation) (13) but substantially increases the test yield by gatekeeping access. ECV_CT_ also seems to track cardiac amyloid burden and, as a result, may also have a future role in monitoring response to therapy, in the same way that CMR-derived ECV can track primary light-chain (AL) cardiac amyloid regression with therapy (40). Normal ECV_CT_ is in the region of 27% (adjusted down by 0.68 ± 1.2% for the averaged, 3-min post-contrast equivalent), which is consistent with the published data in both CT (41) and CMR (15). Patients with lone AS had a higher ECV_CT_ (32% with an averaged, 3-min post-contrast), likely reflecting a degree of myocardial fibrosis (15,42).

We propose different thresholds for onward referral depending on how important grade 1 versus 2 is discovered to be, and whether specificity or sensitivity becomes the priority. A lower threshold of 29% using a 3-min post-contrast acquisition would never miss a case (sensitivity 100%) but would probably result in an unacceptably high referral rate for bone scintigraphy (specificity 19%). A threshold of 31.4% would have a sensitivity of 94% and not miss DPD grade 2 cases but would miss a proportion of DPD grade 1 cases (1 of 5 in our cohort); however, the trade-off is that fewer cases would be referred for an unnecessary DPD (specificity 48%).

Technological developments often result in new insights into established techniques. We were not surprised to find that AS-amyloid was hard to detect based on ECG (e.g., small voltages) or echocardiographic (e.g., reduced MCF) changes because both AS and amyloid can have widely different influences on heart muscle. RBBB being associated with AS-amyloid is interesting and may prove important given that we know it is both common in patients with TAVR and is associated with worse outcome (including higher likelihood of post-TAVR pacemaker insertion) (38). Another interesting finding is that a combination parameter of both ECG and echocardiography, the voltage/mass ratio, performed exceptionally well for amyloid detection compared with parameters derived from just one technique. This is perhaps not surprising as ECV_CT_ and voltage/mass ratio are effectively measuring the same thing: ECV_CT_ measures the proportional size of the water gap between myocytes, and the voltage/mass ratio measures effectively the deficit of electric depolarization from what is expected for a measured wall thickness, which are both measures of myocyte dilution by cardiac amyloid. Unfortunately, Sokolow-Lyon criteria are not validated in patients with bundle branch block (21), either native or from a ventricular paced rhythm, which effectively excluded one-third of our patients. Furthermore, the need to combine information from 2 different measurement techniques is a potential barrier.

### Study limitations

This was a single-center, single-vendor study. ECV_CT_ performance on other vendors has not been assessed but should follow similar methodology. Focal ECV_CT_ elevations were included in the calculated global ECV_CT_, and excluding these areas may increase performance. Our mean patient age was 86 years. Younger cohorts will have possibly lower rates of discovered AS-amyloid. This study is a CT technical development subset of a larger study (including, for example, only those patients who had not already had a CT scan at the time of recruitment); although prevalence and other clinical information informs, this is not the primary focus of this paper. Inline ECV_CT_ software is not yet available, and the work presented here will need to be optimized for integration into the daily CT workflow. Although global longitudinal strain data were included in this study, unfortunately we did not have regional longitudinal strain data available at the time of submission, which may have proven additive in identifying cardiac amyloidosis. The relatively small number of patients with AS-amyloid in this study may also have affected our results.

## Conclusions

Lone AS results in detectable increases in ECV_CT_ compared with control subjects. ECV_CT_ using a low-dose protocol, with a 3-min post-contrast acquisition, can detect AS-amyloid and grade its severity in the TAVR population, and it could be used as a screening tool in those patients already undergoing a clinically indicated CT scan.

Perspectives **COMPETENCY IN MEDICAL KNOWLEDGE:** Pre-TAVR cardiac CT scans can be used to quantify myocardial ECV using a low-dose protocol, with additional baseline and 3-min post-contrast acquisitions.

**TRANSLATIONAL OUTLOOK 1:** ECV_CT_ during routine CT TAVR evaluation can reliably detect AS-amyloid and track the degree of infiltration, offering a potential screening tool in patients already undergoing a clinically indicated CT scan.

**TRANSLATIONAL OUTLOOK 2:** ECV_CT_ is higher in lone AS compared with control subjects due to myocardial fibrosis. Whether this correlates with prognosis in lone AS (as seen in the CMR published data) needs investigation.
