# Supplementary material for: Identifying Cardiac Amyloid in Aortic Stenosis: ECV Quantification by CT in TAVR Patients
Source: JACC Cardiovasc Imaging. 2020 Oct;13(10):2177–89. doi: 10.1016/j.jcmg.2020.05.029 (PMC7536272; doi:10.1016/j.jcmg.2020.05.029)
Supplement: Supplemental Figure 1 and Supplemental Table 1 [file mmc1.docx]

**Supplementary Figure:** Bland-Altman plots comparing 3- and 5-minute post-contrast acquisitions for ECV_CT_ (A) and single shuttle with four shuttle acquisitions (baseline and post-contrast) for ECV_CT_ (B). The 95% limits of agreement are shown in red (-1.70% and 3.06% for **A**; -3.26% and 4.97% for **B**) and the mean difference is shown as the solid black line (0.68% for **A**; 0.85% for **B**). ECV = extracellular volume, ECV_CT_ = extracellular volume quantification using computed tomography.


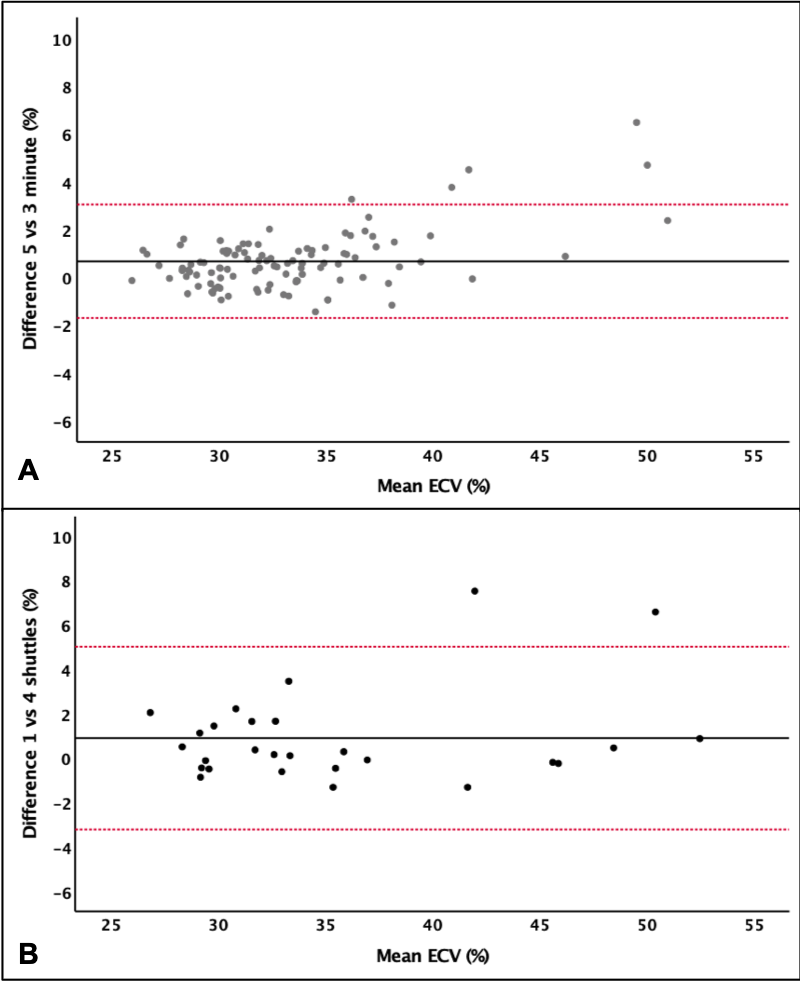


**Supplementary Table:** Variance inflation factors between variables included in the multivariate analysis were overall low (close to 1), suggesting little multicollinearity. ECV_CT_ = extracellular volume quantification by computed tomography, PWd = posterior wall diameter, RBBB = right bundle branch block.

| **Dependent**  **Independent** | **Age** | **ECV_CT_** | **Gender** | **PWd** | **RBBB** |
| --- | --- | --- | --- | --- | --- |
| **Age** | - | 1.142 | 1.106 | 1.059 | 1.118 |
| **ECV_CT_** | 1.056 | - | 1.029 | 1.043 | 1.051 |
| **Gender** | 1.046 | 1.052 | - | 1.078 | 1.064 |
| **PWd** | 1.040 | 1.107 | 1.119 | - | 1.111 |
| **RBBB** | 1.045 | 1.063 | 1.052 | 1.058 | - |
